# Polybrominated Diphenyl Ethers in Human Milk and Serum from the U.S. EPA MAMA Study: Modeled Predictions of Infant Exposure and Considerations for Risk Assessment

**DOI:** 10.1289/EHP332

**Published:** 2016-07-12

**Authors:** Satori A. Marchitti, Suzanne E. Fenton, Pauline Mendola, John F. Kenneke, Erin P. Hines

**Affiliations:** 1 ORISE (Oak Ridge Institute for Science and Education) Fellowship Program, National Exposure Research Laboratory, U.S. Environmental Protection Agency (EPA), Athens, Georgia, USA; 2 National Toxicology Program Laboratory, National Institute of Environmental Health Sciences, National Institutes of Health, Department of Health and Human Services, Research Triangle Park, North Carolina, USA; 3 *Eunice Kennedy Shriver* National Institute for Child Health and Development, Rockville, Maryland, USA; 4 National Exposure Research Laboratory, U.S. EPA, Athens, Georgia, USA; 5 National Center for Environmental Assessment, U.S. EPA, Research Triangle Park, North Carolina, USA

## Abstract

**Background:**

Serum concentrations of polybrominated diphenyl ethers (PBDEs) in U.S. women are believed to be among the world’s highest; however, little information exists on the partitioning of PBDEs between serum and breast milk and how this may affect infant exposure.

**Objectives:**

Paired milk and serum samples were measured for PBDE concentrations in 34 women who participated in the U.S. EPA MAMA Study. Computational models for predicting milk PBDE concentrations from serum were evaluated.

**Methods:**

Samples were analyzed using gas chromatography isotope-dilution high-resolution mass spectrometry. Observed milk PBDE concentrations were compared with model predictions, and models were applied to NHANES serum data to predict milk PBDE concentrations and infant intakes for the U.S. population.

**Results:**

Serum and milk samples had detectable concentrations of most PBDEs. BDE-47 was found in the highest concentrations (median serum: 18.6; milk: 31.5 ng/g lipid) and BDE-28 had the highest milk:serum partitioning ratio (2.1 ± 0.2). No evidence of depuration was found. Models demonstrated high reliability and, as of 2007–2008, predicted U.S. milk concentrations of BDE-47, BDE-99, and BDE-100 appear to be declining but BDE-153 may be rising. Predicted infant intakes (ng/kg/day) were below threshold reference doses (RfDs) for BDE-99 and BDE-153 but above the suggested RfD for BDE-47.

**Conclusions:**

Concentrations and partitioning ratios of PBDEs in milk and serum from women in the U.S. EPA MAMA Study are presented for the first time; modeled predictions of milk PBDE concentrations using serum concentrations appear to be a valid method for estimating PBDE exposure in U.S. infants.

**Citation:**

Marchitti SA, Fenton SE, Mendola P, Kenneke JF, Hines EP. 2017. Polybrominated diphenyl ethers in human milk and serum from the U.S. EPA MAMA Study: modeled predictions of infant exposure and considerations for risk assessment. Environ Health Perspect 125:706–713; http://dx.doi.org/10.1289/EHP332

## Introduction

Polybrominated diphenyl ethers (PBDEs) are persistent flame retardant chemicals used in consumer products. PBDEs were produced in three technical mixtures: pentaBDE (tri-through hexa-brominated congeners), octaBDE (hexa- and hepta-brominated congeners), and decaBDE (primarily deca-brominated BDE-209). PentaBDE and octaBDE, used in polyurethane foam and electronics, respectively, were withdrawn from the U.S. market in 2004. DecaBDE, used in plastics, was phased out in 2013. However, products imported from other countries may still contain PBDEs. In addition, consumer products may be used for many years and cause increased body burdens well beyond the time of manufacture. Animal and *in vitro* studies suggest that PBDEs can cause adverse neurodevelopmental, reproductive, and thyroid effects; however, exposure and health outcomes in humans are not fully understood (Birnbaum 2007; Chao et al. 2011; Eriksson et al. 2001).

The United States was the largest consumer of pentaBDE, and concentrations in samples from the U.S. population (e.g., serum, breast milk) are 10- to 100-fold higher than those reported in Europe or Asia (Marchitti et al. 2013a; Sjödin et al. 2014). Highly lipophilic, these chemicals partition into lipid-rich compartments, including human milk (Marchitti et al. 2013b; Schecter et al. 2003), with the highest exposed population believed to be breastfeeding infants (Johnson-Restrepo and Kannan 2009; Toms et al. 2008). This has led to concern over potential health impacts during sensitive periods of growth and development (Chao et al. 2011; Gascon et al. 2012). Although breastfeeding is known to provide superior benefits to infants and should continue to be encouraged, the lack of nationally representative breast milk data has limited our understanding of the concentrations of environmental chemicals in breast milk and our ability to assess infant exposures (Lehmann et al. 2014). One approach involves developing models that extrapolate chemical concentrations from a surrogate or alternative matrix, such as maternal serum, to predict levels in breast milk using chemical-specific milk:serum partitioning ratios. The United States has several years of nationally representative serum data on many persistent chemicals from its National Health and Nutrition Examination Survey (NHANES), and these surveys are continuing to produce such data (http://www.cdc.gov/exposurereport/). However, limited partitioning information exists for environmental chemicals, and the use of PBDE concentrations in maternal blood as a surrogate for predicting maternal and infant exposure is still a developing field. The U.S. EPA (Environmental Protection Agency) Methods Advancement for Milk Analysis (MAMA) Study was initiated to better understand environmental chemical concentrations in women and the factors that influence chemical partitioning and infant exposure (Hines et al. 2009, 2015). The objectives of the present study were to measure and compare concentrations of PBDEs in paired samples of serum and milk donated by women in the MAMA Study. Serum was evaluated as a surrogate for predicting breast milk concentrations and infant exposure, and sources of PBDE exposure were explored using demographic and lifestyle information.

## Methods

### Human Subjects

A convenience sample of 34 healthy, breastfeeding women (21–39 years) were recruited by Westat Inc. (Chapel Hill, NC) and asked to provide informed consent before participating in this study, as described (Hines et al. 2009). Milk and serum samples were collected at 2–7 weeks (first visit) and 3–4 months (second visit) postpartum between December 2004 and July 2005. A demographic and lifestyle questionnaire was administered at the first visit (see Supplemental Material, “First Visit Questionnaire, U.S. EPA MAMA Study”). Human subject participation was approved by the institutional review boards of the University of North Carolina (IRB 03-EPA-207) and the Centers for Disease Control and Prevention (CDC; IRB 3961).

### Sample Collection

Women were asked to fast for 1.5 hr before arrival. Milk (90 mL into PBDE-free polypropylene tubes) was collected using a breast pump (Medela). At the same visit, blood (20 mL) was collected via venipuncture into nonheparinized glass vacutainer tubes (Becton Dickinson). Blood was clotted (1 hr) and spun at 3,000 rpm (15 min) to collect serum. Samples were shipped on dry ice to the CDC (Atlanta, GA) and stored at –20°C.

### Serum and Milk Analysis

Nine PBDE congeners [2,4,4´-tribromodiphenyl ether (BDE-28), 2,2´,4,4´-tetrabromodiphenyl ether (BDE-47), 2,3´,4,4´-tetrabromodiphenyl ether (BDE-66), 2,2´,3,4,4´-pentabromodiphenyl ether (BDE-85), 2,2´,4,4´,5-pentabromodiphenyl ether (BDE-99), 2,2´,4,4´,6-pentabromodiphenyl ether (BDE-100), 2,2´,4,4´,5,5´-hexabromodiphenyl ether (BDE-153), 2,2´,4,4´,5,6´-hexabromodiphenyl ether (BDE-154), and 2,2´,3,4,4´,5´,6-heptabromodiphenyl ether (BDE-183)] were measured in serum and milk (ng/g lipid), as described (Sjödin et al. 2004a, 2004b). 2,2´,4-Tribromodiphenyl ether (BDE-17) was measured in serum but not in milk. In addition to the PBDEs, the brominated flame retardant 2,2´,4,4´,5,5´-hexabromobiphenyl (BB-153) was also measured as part of the standard CDC panel of flame retardants. Serum samples were extracted, denatured, and analyzed using gas chromatography isotope dilution high-resolution mass spectrometry (GC-ID-HRMS) with a MAT95XP instrument (Thermo Electron). Serum lipid concentration was determined using available kits (Roche Diagnostics). Milk samples underwent solid-phase dispersion onto diatomaceous earth and were dried by pressurized nitrogen; lipids and target analytes were extracted with dichloromethane. Milk lipids were determined gravimetrically, and analysis performed by GC-ID-HRMS. Blank (*n* = 3) and quality control (*n* = 3) samples were included with each set of unknown specimens (batch *n ≤* 24).

Laboratory quality assurance practices were regularly monitored (Sjödin et al. 2004a, 2004b). The limits of detection (LOD) in serum and milk were defined as three times the standard deviation of the blank samples; in the absence of a signal in the blank samples, the LOD was determined to be the lowest calibration standard having a signal-to-noise ratio > 10. LOD values varied for each PBDE and were dependent on sample size and potential interferences detected in blank samples.

### Predictions of Milk PBDE Concentrations and Infant Exposure

Exposure models for predicting milk PBDE concentrations from serum concentrations have been developed (Marchitti et al. 2013b). These models rely on congener-specific predictions of PBDE milk:serum partitioning ratios and have been validated but not extensively tested (see Supplemental Material, “Additional modeling information”). To test them, model equations were applied to serum PBDE concentrations from MAMA Study participants to calculate predicted milk PBDE concentrations, which were then compared to observed values. Models were then applied to NHANES PBDE serum data available from 2003–2008 for women 20–39 years of age to obtain predicted representative milk PBDE concentrations for the U.S. population. NHANES serum PBDE concentrations represented individual samples (2003–2004) or pooled samples (2005–2006 and 2007–2008) for different ethnicities (non-Hispanic white, non-Hispanic black, and Mexican American); weighted arithmetic means (ng/g lipid) are intended to be representative of the U.S. population (Sjödin et al. 2014).

From predicted milk concentrations, average daily intakes of PBDEs in U.S. infants (DI_i_; ng/kg bw/day) were calculated using the formula: *DI_i_* = *C_m_FMb* (Johnson-Restrepo et al. 2007), where *C_m_* is our predicted mean PBDE concentration in U.S. breast milk (ng/g lw), *F* is the average lipid content of breast milk (4.0 g lipid/100 g milk), and *Mb* is the average daily consumption of milk [150 mL/kg/day (milk density 1.03 g/mL)] for infants age birth to < 1 month, as recommended by the *Exposure Factors Handbook* of the U.S. EPA (2011).

### Statistics

PBDE concentrations < LOD were assigned a value equal to the LOD divided by the square root of 2. To determine PBDE milk:serum partitioning ratios, only participants with both serum and milk concentrations > LOD were used (*n* = 5–34 women) for individual PBDEs. Correlations and statistical comparisons were performed only for compounds detected in ≥ 50% of samples. Spearman correlation coefficients (*r*_s_) assessed the relationship between milk and serum PBDE concentrations. Statistical comparisons were performed using paired *t*-tests.

Questionnaire data were examined for associations with PBDE concentrations; categorical variables were assessed using one-way analysis of variance while continuous variables were assessed with Spearman correlations. Given the exploratory nature of the study, adjustments were not made for multiple comparisons. Analyses were conducted using SAS Enterprise Guide 4.1 (SAS Institute Inc., Cary, NC). Significance was denoted at *p* ≤ 0.05.

## Results

### Concentrations of PBDEs in Serum

**Figure 1:**
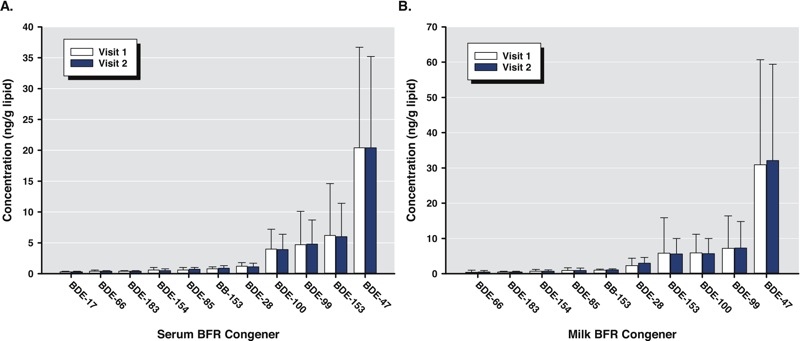
PBDE concentrations (ng/g lipid) in serum (*A*) and milk (*B*) at visits 1 and 2 from women in the U.S. EPA MAMA Study, 2004–2005. Data presented are geometric means ± 95% confidence intervals (*n* = 18–34). BFR, brominated flame retardant.

Serum PBDE detection rates ranged from 3–100% (Table 1). BDE-47 was measured at the highest concentration (median, 18.6 ng/g lipid). BDE-99, BDE-100, and BDE-153 were measured at median concentrations of 3.6, 3.9, and 5.5 ng/g lipid, respectively. Remaining analytes were measured at median concentrations of ≤ 1.1 ng/g lipid. Serum PBDE concentrations did not differ significantly between visits (Figure 1).

**Table 1:** Concentrations of PBDEs in serum (ng/g lipid) from women in the U.S. EPA MAMA Study.

PBDE	Weeks postpartum^*a*^	*n*	% detect	LOD (ng/g lipid)	GM (95% CI)	5th percentile	25th percentile	Median	75th percentile	95th percentile	Range
BB-153	2–7	33	70	0.3–0.5	0.8 ± 0.3	0.3	0.4	1.0	1.7	2.5	0.2–4.6
	12–16	30	67	0.3–0.7	0.9 ± 0.4	0.2	0.4	1.0	1.5	3.4	0.2–5.5
BDE-17	2–7	34	15	0.3–0.5	0.3 ± 0.1	0.3	0.3	0.3	0.4	0.8	0.2–1
	12–16	30	3	0.3–0.7	0.3 ± 0.1	0.2	0.3	0.4	0.4	0.5	0.2–1.1
BDE-28	2–7	34	91	0.3–1	1.2 ± 0.6	0.4	0.8	1.1	1.8	7.1	0.3–8.2
	12–16	30	63	0.3–1.3	1.1 ± 0.6	0.4	0.8	1.0	1.7	6.5	0.3–6.6
BDE-47	2–7	34	100	0.4–6	20.4 ± 16.3	4.8	12.2	18.6	28.7	173.0	4.5–227.0
	12–16	30	100	0.3–7.9	20.4 ± 14.8	6.7	12.1	16.8	28.2	111.0	5.4–222.0
BDE-66	2–7	34	38	0.3–0.5	0.4 ± 0.2	0.3	0.3	0.4	0.5	1.9	0.3–2.4
	12–16	30	13	0.3–0.7	0.4 ± 0.1	0.2	0.3	0.4	0.4	1.1	0.2–2.1
BDE-85	2–7	34	53	0.3–0.9	0.6 ± 0.4	0.2	0.3	0.5	0.7	4.5	0.2–5.3
	12–16	30	47	0.3–1.2	0.7 ± 0.3	0.3	0.4	0.6	0.9	2.6	0.3–5.1
BDE-99	2–7	34	97	0.3–3.2	4.7 ± 5.4	1.0	2.1	3.6	6.8	61.9	1–62.7
	12–16	30	83	0.4–4.2	4.8 ± 3.9	1.7	2.8	3.9	7.8	30.9	1.2–57.6
BDE-100	2–7	34	97	0.3–0.9	4.0 ± 3.2	0.9	2.3	3.9	5.7	29.3	0.4–39.1
	12–16	30	100	0.3–1.2	3.9 ± 2.5	1.1	2.2	4.1	4.9	28.5	0.9–28.9
BDE-153	2–7	34	97	0.3–1.4	6.2 ± 8.4	1.0	2.6	5.5	15.3	63.5	1.0–134.0
	12–16	30	100	0.3–1.9	6.0 ± 5.4	1.4	2.9	5.1	13.6	39.7	1.3–68.8
BDE-154	2–7	34	53	0.3–0.5	0.6 ± 0.4	0.2	0.3	0.4	0.7	4.2	0.2–5.2
	12–16	30	53	0.3–0.7	0.5 ± 0.3	0.3	0.4	0.5	0.6	2.6	0.2–3.7
BDE‑183	2–7	34	29	0.3–0.5	0.4 ± 0.1	0.3	0.3	0.4	0.4	0.8	0.2–0.9
	12–16	29	14	0.3–0.7	0.4 ± 0.1	0.2	0.3	0.4	0.4	0.7	0.2–1.1
ΣPBDEs	2–7	34	ND	ND	43.2 ± 30.7	13.6	24.7	38.4	63.5	327.0	10.5–369.0
	12–16	30	ND	ND	42.8 ± 23.5	15.3	26.5	41.0	57.6	172.5	11.7–341.1
Abbreviations: CI, confidence interval; GM, geometric mean; ND, not determined. ^***a***^Visit 1, 2–7 weeks postpartum; visit 2, 12–16 weeks postpartum.											

### Concentrations of PBDEs in Human Milk

Milk PBDE detection rates ranged from 26–100% (Table 2). BDE-47 was measured at the highest concentration (median, 31.5 ng/g lipid). BDE-99, BDE-100, and BDE-153 were measured at median concentrations of 6.0, 5.3, and 5.0 ng/g lipid, respectively. The remaining analytes were measured at median concentrations of ≤ 2.4 ng/g lipid. Milk PBDE concentrations varied for individuals between visits (range, –89% to 700%), however, median changes (range, –4% to 6%) were not significantly different from zero, and no significant differences in milk concentrations between visits were detected (Figure 1).

**Table 2:** Concentrations of PBDEs in milk (ng/g lipid) from women in the U.S. EPA MAMA Study.

PBDE	Weeks postpartum^*a*^	*n*	% detect	LOD (ng/g lipid)	GM (95% CI)	5th percentile	25th >percentile	Median	75th percentile	95th percentile	Range
BB-153	2–7	34	74	0.2–0.9	1.0 ± 0.3	0.3	0.6	1.0	1.6	3.1	0.3–3.4
	12–16	30	87	0.1–2.3	1.1 ± 0.3	0.3	0.6	1.3	1.8	3.3	0.2–4.4
BDE-28^*b*^	2–7	18	100	0.2–0.9	2.3 ± 2.1	0.8	1.5	2.0	2.9	14.7	0.6–17.6
	12–16	22	100	0.1–2.3	3.0 ± 1.6	1.3	1.7	2.4	5.1	14.2	1.3–15.2
BDE-47	2–7	34	100	0.8–3.1	30.9 ± 29.8	6.0	15.7	31.5	49.2	236.4	5.9–462.5
	12–16	30	100	0.5–8.1	32.1 ± 27.3	7.8	20.2	28.2	49.8	226.0	6.1–394.5
BDE-66^*b*^	2–7	18	39	0.2–0.9	0.4 ± 0.6	0.2	0.3	ND	0.4	3.1	0.1–4.9
	12–16	22	73	0.1–2.3	0.5 ± 0.4	0.1	0.3	0.4	0.6	2.2	0.1–4.0
BDE-85	2–7	34	59	0.2–0.9	0.9 ± 0.8	0.2	0.4	0.8	2.0	6.4	0.2–10.3
	12–16	30	73	0.1–2.3	0.9 ± 0.7	0.3	0.5	0.8	1.5	5.6	0.2–8.8
BDE-99	2–7	34	100	0.7–2.6	7.2 ± 9.2	1.9	3.8	6.0	11.0	84.2	1.3–128.0
	12–16	30	100	0.4–6.6	7.3 ± 7.5	2.2	4.1	6.7	11.0	68.2	1.5–104.5
BDE-100	2–7	34	100	0.2–0.9	5.9 ± 5.3	1.6	3.1	5.3	7.3	51.9	0.9–65.2
	12–16	30	100	0.1–2.3	5.7 ± 4.3	1.3	3.6	4.9	7.8	45.0	1.0–47.3
BDE-153	2–7	33	100	0.3–1.2	5.8 ± 10.1	1.1	2.0	5.0	15.8	58.7	0.7–163.4
	12–16	30	100	0.2–3.2	5.6 ± 4.4	1.0	2.8	5.2	13.6	39.1	0.9–45.1
BDE-154	2–7	34	65	0.2–0.9	0.7 ± 0.5	0.2	0.4	0.6	1.3	5.4	0.2–6.8
	12–16	30	77	0.1–2.3	0.7 ± 0.4	0.3	0.4	0.6	0.9	4.0	0.2–4.5
BDE‑183	2–7	34	26	0.2–0.9	0.5 ± 0.2	0.2	0.3	ND	0.6	1.5	0.2–2.9
	12–16	30	27	0.1–2.3	0.5 ± 0.2	0.2	0.3	ND	0.7	1.8	0.2–1.9
ΣPBDEs	2–7	34	ND	ND	57.1 ± 50.1	17.6	32.8	50.8	77.2	453.1	10.3–676.6
	12–16	30	ND	ND	59.4 ± 41.4	18.1	38.9	54.2	80.1	300.0	10.7–577.0
Abbreviations: CI, confidence interval; GM, geometric mean; ND, not determined. ^***a***^Visit 1, 2–7 weeks postpartum; visit 2, 12–16 weeks postpartum. ^***b***^Concentrations of BDE-28 and BDE-66 are reported from a subset of the study population (*n* = 18–22).											

### PBDE Milk:Serum Partitioning

No significant differences in milk:serum partitioning ratios were found between visits, so data were combined. BDE-28 demonstrated the highest milk:serum partitioning ratio (2.1; Table 3). Milk concentrations of tri- through hexa-brominated congeners were significantly higher than corresponding serum concentrations, resulting in milk:serum partitioning ratios significantly > 1. Predicted milk:serum ratios using models in the study by Marchitti et al. (2013b) fell within confidence intervals of those observed in MAMA participants, except for BDE-100 and BDE-28, where observed values were slightly higher by 7–16% (Table 3).

**Table 3:** Median PBDE Milk:serum partitioning ratios and chemical properties.

PBDE	Observed^*a*^ milk:serum ratios	Predicted^*b*^ milk:serum ratios	Chemical properties		
			Mol wt	No. Br atoms	Log K_ow_^*c*^
BDE-28*	2.1 ± 0.2 (*n* = 25)	1.4 ± 0.2	406.9	3	5.94
BDE-47*	1.7 ± 0.1 (*n* = 34)	1.5 ± 0.1	485.8	4	6.81
BDE-66*	1.9 ± 0.5 (*n* = 5)	ND	485.8	4	NR
BDE-85*	1.4 ± 0.5 (*n* = 17)	1.3 ± 0.2	564.7	5	NR
BDE-99*	1.4 ± 0.1 (*n* = 34)	1.4 ± 0.2	564.7	5	7.32
BDE-100*	1.5 ± 0.1 (*n* = 34)	1.3 ± 0.0	564.7	5	7.24
BB-153	1.0 ± 0.1 (*n* = 24)	ND	627.6	6	9.10
BDE-153	0.9 ± 0.1 (*n* = 33)	0.9 ± 0.0	643.6	6	7.90
BDE-154	1.2 ± 0.4 (*n* = 20)	0.8 ± 0.1	643.6	6	7.82
BDE-183	0.6 ± 0.2 (*n* = 6)	ND	722.5	7	8.27
Abbreviations: Br, bromine; mol wt, molecular weight; ND, not determined; NR, not reported. ^***a***^Milk:serum ratios were determined from MAMA participants with both milk and serum measurements > LOD (*n* = 5–34 women, depending on PBDE). Data from both visits were combined. ^***b***^Milk:serum ratios were predicted using models developed by Marchitti et al. (2013b). ^***c***^Source: Braekevelt et al. 2003; Hardy 2002. *Analytes with milk:serum partitioning ratios significantly > 1 for MAMA Study participants (*p* < 0.02).					

### Correlations

Milk PBDE concentrations and serum PBDE concentrations were highly correlated for individual PBDEs and Σ_6_PBDE (*r*_s_ = 0.57–0.97; *p* < 0.0001; *n* = 62–63 samples). PBDE concentrations were not correlated with most questionnaire variables including weeks postpartum, parity, prior breastfeeding, body mass index (BMI), diet, or age of furniture in the home. However, BB-153 concentrations (milk and serum) were moderately correlated (as defined as *r_s_* = 0.25–0.5) with maternal age [*r_s_* = 0.35–0.47, *p* < 0.01 (see Table S1)] and inversely correlated (milk only) with vehicle age [*r_s_* = –0.30, *p* < 0.02 (see Table S2)]. Concentrations of some PBDEs were inversely correlated with both maternal age [*r_s_* = –0.26 to –0.40, *p* < 0.05 (see Table S1)] and home age [*r_s_* = –0.25 to –0.36, *p* < 0.05 (see Table S3)], but positively correlated with vehicle age [*r_s_* = 0.29–0.45, *p* < 0.05 (see Table S2)].

### Modeled Predictions of Milk PBDE Concentrations

**Figure 2:**
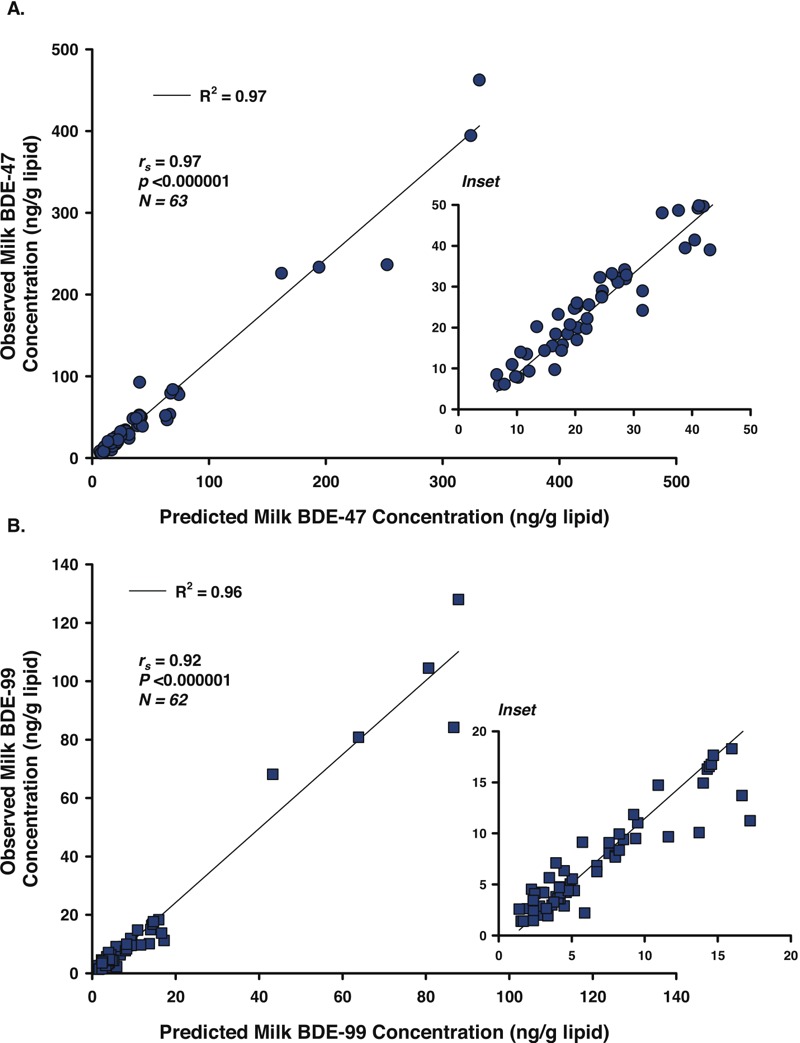
Predicted milk BDE-47 (*A*) and BDE-99 (*B*) concentrations versus those observed in MAMA Study participants. Solid lines are the least-squares fit of predicted and observed concentrations [*R* ^2^ = 0.97, slope = 1.24 (*A*); *R* ^2^ = 0.96, slope = 1.27 (*B*)]. Spearman correlation coefficients (*r*_s_) and related *p-*values are shown.

Models developed by Marchitti et al. (2013b) were tested using MAMA data; predicted and observed milk PBDE concentrations were highly correlated (Figure 2; see Figure S1). Applying the models to NHANES serum PBDE data for women of childbearing age (20–39 years) (Sjödin et al. 2014) allowed us to predict national concentrations of PBDEs in U.S. breast milk for three time periods [2003–2004, 2005–2006, and 2007–2008 (Table 4)]. Mean milk PBDE concentrations for MAMA participants, primarily non-Hispanic whites, were within confidence intervals for concentrations predicted for non-Hispanic whites in NHANES 2003–2005.

### Daily Infant Intakes

Predicted mean U.S. milk PBDE concentrations (Table 4) were used to estimate average daily infant intakes (DI_i_; ng/kg/day) using the formula *DI_i_* = *C_m_FMb*, as described in “Methods.” For 2007–2008, estimated PBDE intakes for U.S. infants were 274–282 ng/kg/day (BDE-47), 54–65 ng/kg/day (BDE-99), 46–57 ng/kg/day (BDE-100), and 61–66 ng/kg/day (BDE-153), depending on ethnic group (Table 4). Estimated PBDE intakes for MAMA participant infants were 331 ng/kg/day (BDE-47), 89 ng/kg/day (BDE-99), 65 ng/kg/day (BDE-100), and 89 ng/kg/day (BDE-153) (Table 4).

**Table 4:** Predicted U.S. milk PBDE concentrations and estimated daily infant intakes.

PBDE	Year	Predicted U.S. milk^*a*^**(ng/g lipid)			Estimated daily intakes for U.S. infants^*b*^ (ng/kg/day)			MAMA study participants^*c*^	
		NHW	NHB	MA	NHW	NHB	MA	Milk (ng/g lipid)	Infant^*d*^ (ng/kg/day)
BDE-47	>2003–2004	59.4 ± 24.5	78.1 ± 37.4	57.8 ± 29.1	356 ± 147	469 ± 224	347 ± 175	55.2	331
	2005–2006	73.0 ± 54.5	89.1 ± 65.4	44.4 ± 13.0	438 ± 327	535 ± 392	266 ± 78
	2007–2008	47.0 ± 22.3	47.0 ± 12.0	45.6 ± 29.9	282 ± 134	282 ± 72	274 ± 179
BDE-99	2003–2004	15.4 ± 8.1	23.1 ± 15.7	11.5 ± 4.5	92 ± 49	139 ± 94	69 ± 27	14.9	89
	2005–2006	18.8 ± 15.4	24.5 ± 22.4	9.1 ± 2.2	113 ± 92	147 ± 134	55 ± 13
	2007–2008	10.8 ± 3.1	10.1 ± 3.2	9.0 ± 7.1	65 ± 19	61 ± 19	54 ± 43
BDE-100	2003–2004	12.7 ± 5.0	10.8 ± 5.0	8.2 ± 3.8	76 ± 30	65 ± 30	49 ± 23	10.9	65
	2005–2006	12.5 ± 7.2	14.7 ± 14.6	6.8 ± 2.4	75 ± 43	88 ± 88	41 ± 14
	2007–2008	7.7 ± 2.6	9.3 ± 3.7	9.5 ± 5.9	46 ± 16	56 ± 22	57 ± 35
BDE‑153	2003–2004	16.3 ± 5.8	7.8 ± 3.2	5.6 ± 2.2	97 ± 35	47 ± 19	34 ± 13	14.8	89
	2005–2006	10.3 ± 3.7	8.9 ± 4.0	6.2 ± 3.5	62 ± 22	53 ± 24	37 ± 21
	2007–2008	11.0 ± 3.3	10.2 ± 3.8	10.3 ± 5.8	66 ± 20	61 ± 23	62 ± 35
Abbreviations: MA, Mexican American; NHB, non-Hispanic black; NHW, non-Hispanic white. ^***a***^Predicted U.S. milk PBDE concentrations were determined by applying congener-specific models (Marchitti et al. 2013b) to NHANES mean serum data for women of child-bearing age [20–39 years (Sjödin et al. 2014)]. ^***b***^Estimated intakes for U.S. infants were calculated from predicted U.S. milk PBDE concentrations (this table) using the formula *DI*_*i*_* = C*_*m*_*FMb*. ^***c***^MAMA Study (2004–2005) mean milk concentrations (visit 1) from 34 women (21–39 years); 85% NHW, 9% NHB, and 3% MA. ^***d***^Infant intakes for MAMA Study participants were estimated from MAMA milk concentrations (this table) using the formula *DI*_*i*_* = C*_*m*_*FMb*.									

## Discussion

As concentrations of other persistent chemicals, such as PCBs and dioxins, in human serum and milk have decreased in recent decades, concentrations of PBDEs were found to be increasing, and human exposures are expected to continue for many years (Schecter et al. 2010; Sjödin et al. 2014). U.S. women of childbearing age have among the world’s highest milk PBDE concentrations, which has led to concern over potential health impacts to breastfeeding infants (Bradman et al. 2007; Park et al. 2011). However, a lack of comprehensive data on breast milk concentrations of PBDEs in the United States has precluded our ability to conduct more complete exposure assessments. Although national data on chemicals in milk would be preferred, nationally representative data on hundreds of chemicals in serum is available and estimating U.S. population-level breast milk concentrations from these serum concentrations using congener-specific milk:serum partitioning information would be desirable.

Historically, in the absence of data on milk:serum partitioning, risk assessors have assumed that, at steady state, lipophilic and persistent chemicals partition to lipid stores in the body equally, such that the lipid-based concentration in one matrix, such as serum, can be assumed to be equal to the lipid-based concentration in a different matrix, such as breast milk. The science has since advanced, and recent studies suggest this approach is too simplistic and does not accurately take into account what is now understood about the complexities of chemical partitioning (LaKind et al. 2009; Mannetje et al. 2012; Marchitti et al. 2013a, 2013b). Various factors and physicochemical properties have been shown to influence partitioning between maternal serum and breast milk including molecular weight, molecular size, steric hindrance, lipophilicity, and halogenation (Needham et al. 2011). Thus, partitioning can vary substantially among different congeners of the same chemical class, and those congeners with greater distribution into breast milk should be evaluated more prudently when determining infant exposures (Mannetje et al. 2012; Marchitti et al. 2013b). To measure accurate partitioning ratios, careful consideration should be given to study design with milk and serum samples taken sufficiently postpartum (after the milk supply has been adequately established), and as close in time as possible from the same woman (i.e., within 1–2 hr). Few studies met these criteria prior to 2006; however, recent quality data available for PBDEs (LaKind et al. 2009; Schecter et al. 2006, 2010) were used to develop the first congener-specific models and approach for estimating nationally representative breast milk PBDE concentrations in the U.S. population from NHANES serum concentrations (Marchitti et al. 2013b). Although these models were initially validated and found to be highly predictive, they had yet to be rigorously tested or extended to more recent NHANES data sets. In this study, we have presented for the first time serum and milk concentrations of PBDEs from paired samples taken from women enrolled in the U.S. EPA MAMA Study, which followed quality partitioning data guidelines, and the use of these data to further test and expand these exposure models.

Serum and milk concentrations of PBDEs in MAMA Study participants were highly correlated. Consistent with other reports (Marchitti et al. 2013b; Schecter et al. 2010), BDE-47 was present at the highest concentrations in milk and serum, and lower brominated PBDEs demonstrated higher milk:serum partitioning ratios than higher brominated congeners (PBDE milk:serum ratio: 3 Br > 4 Br > 5 Br >> 6 Br >> 7 Br). Although PBDE log K_ow_ values generally increase with increasing halogenation, steric hindrance and overall size of the higher brominated congeners may substantially limit their ability to distribute into breast milk. A recent review of available chemical partitioning data from 13 studies for persistent lipophilic organic chemicals, including PCDD/Fs (polychlorinated dibenzo-*p*-dioxins and furans), PCBs (polychlorinated biphenyls), PBDEs, and organochlorine pesticides, reported that mean partitioning ratios can vary by approximately 30-fold among a comprehensive list of specific congeners (Mannetje et al. 2012). Although most congeners appeared to have partitioning ratios that ranged more narrowly, albeit with congener-specific differences, ratios determined for the higher halogenated congeners of each chemical class (e.g., the deca-brominated BDE-209) suggest they have a very limited capacity to distribute into breast milk (Mannetje et al. 2012).

Exposure models developed for predicting milk PBDE concentrations from serum concentrations (Marchitti et al. 2013b) demonstrated high accuracy and predictability when tested against MAMA serum and milk data. Further application of these models to more recent NHANES serum data (through 2008) suggest that BDE-47, BDE-99, and BDE-100 are declining in U.S. breast milk for non-Hispanic white and non-Hispanic black populations, although these results were not statistically significant. For some populations, however, milk concentrations of BDE-100 (Mexican Americans) and BDE-153 (all ethnic groups) may be increasing, which may reflect unknown exposure sources related to diet or lifestyle. Predicted U.S. infant PBDE intakes are expected to follow the same trends, and as of the 2007–2008 time period, estimated daily infant intakes were below threshold reference doses (RfDs) for BDE-99 [RfD, 100 ng/kg body weight (bw)/day (U.S. EPA 2008a)] and BDE-153 [RfD, 200 ng/kg bw/day (U.S. EPA 2008b)] but above that suggested for BDE-47 [RfD, 100 ng/kg bw/day (U.S. EPA 2008c)]. Other studies have reported similar findings (Johnson-Restrepo et al. 2007; Park et al. 2011). Studies evaluating exposure to concentrations of PBDEs in breast milk in the highest quartiles (3rd and 4th) suggest subtle associations between early-life PBDE exposure and increased anxiety, withdrawal, and activity/impulsivity behaviors in early childhood (Adgent et al. 2014; Hoffman et al. 2012). However, improvement in certain adaptive behaviors and cognitive outcomes were also associated, suggesting that the positive factors associated with breastfeeding may mitigate some of the potentially adverse outcomes associated with PBDEs.

RfDs are determined by the U.S. EPA’s Integrated Risk Information System (IRIS) and are estimates of daily oral human exposure to chemicals that are not likely to cause harmful effects during a lifetime. These daily values are based on dose–response assessments from available studies; uncertainty may span orders of magnitude and is based on the design of the studies providing the dose–response data and how well those studies model a lifetime human exposure scenario. The RfD for BDE-47 (100 ng/kg bw/day) was derived from dose–response data for behavioral impairments observed in adult male mice following a single oral dose administered 10 days after birth, which represents a period of maximum vulnerability for mouse brain development (Eriksson et al. 2001). Calculation of this RfD included the application of an uncertainty factor of 3,000, accounting for *a*) the possibility that some people may be more sensitive to the effects of BDE-47 than others, *b*) the possibility that humans may be more sensitive than mice, *c*) the fact that the study administered only a single dose of the chemical whereas human exposure may be expected to occur on a daily basis over a prolonged period of time, and *d*) deficiencies in the toxicological database for BDE-47, which, at the time the RfD was derived, lacked prenatal developmental neurotoxicity studies, reproductive toxicity studies, and standard chronic or subchronic studies of systemic toxicity (U.S. EPA 2008c). Although RfDs are derived for one chemical at a time, humans are exposed concomitantly to complex mixtures of chemicals, including PBDE congeners; thus, an active area of research is the collective impacts of chemical combinations on human health.

Milk PBDE concentrations from MAMA participants (residing in North Carolina) were comparable to those reported from many U.S. states but were substantially (58–76%) higher than those from New Hampshire, Massachusetts, and Texas (Table 5). This may reflect differences in study demographics or regional differences in PBDE use or legislation. It is unclear whether concentrations of PBDEs in breast milk decrease with breastfeeding duration (i.e., depuration). One study (*n* = 18) reported a significant decrease (12–18%) in milk PBDE concentrations during the first 6 months postpartum (Hooper et al. 2007), and another study (*n* = 303) reported a significant increase (18%) in milk BDE-153 concentrations from 3 to 12 months postpartum (Daniels et al. 2010). Other studies have reported no consistent pattern in milk PBDE concentrations over time (Dunn et al. 2010; LaKind et al. 2009). The median interval between milk collections in MAMA participants was 8.1 weeks, and no evidence of depuration was observed.

**Table 5:** Median milk concentrations (ng/g lipid) from U.S. studies, 2002–2006.

Study	State	Year	*n*	BDE-47	BDE-99	BDE-100	BDE-153
U.S. EPA MAMA study^*a*^	NC	2004–2005	34	31.5	6.0	5.3	5.0
Daniels et al. 2010	NC	2004–2006	303	28.0	5.0	5.0	6.0
Dunn et al. 2010	NH	2005–2006	40	13.4	2.0	2.5	5.0
Wu et al. 2007	MA	2004–2005	46	13.9	2.4	2.4	3.1
Johnson-Restrepo et al. 2007	MA	2004	38	7.7	1.5	0.5	1.1
LaKind et al. 2009	PA	2004–2005	10	26.0	4.6	4.0	3.5
LaKind et al. 2008	PA	2004–2006	19	24.0	3.8	3.6	3.4
Schecter et al. 2003	TX	2002	47	18.4	5.7	2.9	2.0
Schecter et al. 2006	TX	2003	11	10.0	2.0	2.1	2.4
Park et al. 2011	CA	2003–2005	82	29.7	6.4	5.7	6.3
She et al. 2007	PNW^*b*^	2003	40	27.8	5.4	5.3	4.8
^***a***^Visit 1 median values. ^***b***^PNW, Pacific Northwest (MT, OR, and WA).							

### Study Limitations

The MAMA Study was limited to a small convenience sample of primarily non-Hispanic white, well-educated mothers. Preliminary evaluation of demographic and lifestyle variables indicated that, as in other studies (Petreas et al. 2003), concentrations of PBDEs in milk and serum from MAMA participants decreased with maternal age. PBDE concentrations were not associated with either dietary preferences or parity, which is similar to other findings (Guvenius et al. 2003); however, our survey questions on diet may have been too limited and did not account for many subcategories such as various forms of dairy (e.g., cheese, ice cream, yogurt) nor discriminate among alternative types of milk (e.g., soy, almond milk). In addition, approximately half of the MAMA participants were first-time mothers, which limited our analysis of parity. Moreover, because of the small sample size, correlations between PBDE concentrations and demographics may be ancillary observations, and further research is necessary. This need is not unique to PBDEs. Indeed, the lack of nationally representative data for environmental chemicals in breast milk in general has substantially limited exposure assessments for infants and young children (Lehmann et al. 2014). As a solution to this problem, some countries (e.g., Sweden and Germany) have conducted breast milk monitoring programs; however, no such program exists in the United States. As a sustainable alternative, we have developed models to predict breast milk PBDE concentrations based on serum concentrations (Marchitti et al. 2013b). This study further validated these models and applied them to NHANES serum data from 2003–2008 to obtain nationally representative estimates of PBDE concentrations in breast milk in the U.S. population over time. From these data, we estimated daily infant intakes of PBDEs meant to be representative for U.S. breastfeeding infants. Before this study, no national intake data for U.S. infants were available. However, caution should be used when interpreting our results because two extrapolations have been performed in this study: *a*) from U.S. women of childbearing age in the NHANES survey to U.S. lactating women, and *b*) from U.S. breast milk concentrations to U.S. daily infant intakes. These extrapolations may increase uncertainty; however, until data are available to allow for more direct evaluations of early-life exposures, such extrapolations expedite the development of methods and tools to improve risk assessment for infants and young children.

## Conclusions

Maternal serum, in conjunction with milk:serum partitioning models, appears to be an acceptable surrogate for predicting PBDE concentrations in human milk. Using NHANES serum data, concentrations of most PBDEs in U.S. breast milk appear to be declining but concentrations of BDE-153 may be rising, which may reflect an unknown source related to diet or lifestyle. Breastfeeding confers extensive developmental benefits to infants and should continue to be recommended despite the presence of environmental chemicals. However, continued research of the molecular complexities of chemical partitioning is necessary for performing accurate infant exposure and risk assessments.

## Supplemental Material

(196 KB) PDF
