# Supplementary material for: Polybrominated Diphenyl Ethers in Human Milk and Serum from the U.S. EPA MAMA Study: Modeled Predictions of Infant Exposure and Considerations for Risk Assessment
Source: Environ Health Perspect. 2016 Jul 12;125(4):706–13. doi: 10.1289/EHP332 (PMC5381986; doi:10.1289/EHP332)
Supplement: (196 KB) PDF [file EHP332.s001.acco.pdf]

Environ Health Perspect
DOI: 10.1289/EHP332

Note to readers with disabilities: *EHP* strives to ensure that all journal content is accessible to all readers. However, some figures and Supplemental Material published in *EHP* articles may not conform to 508 standards due to the complexity of the information being presented. If you need assistance accessing journal content, please contact ehp508@niehs.nih.gov. Our staff will work with you to assess and meet your accessibility needs within 3 working days.

Supplemental Material

Polybrominated Diphenyl Ethers in Human Milk and Serum from the US EPA MAMA Study: Modeled Predictions of Infant Exposure and Considerations for Risk Assessment

Satori A. Marchitti, Suzanne E. Fenton, Pauline Mendola, John F. Kenneke, and Erin P. Hines

Table of Contents

First Visit Questionnaire, U.S. EPA MAMA Study
Table S1. Spearman correlation between maternal age and BFR concentrations
Table S2. Spearman correlation between vehicle age and BFR concentrations
Table S3. Spearman correlation between home age and BFR concentrations
Figure S1. Predicted milk BFR concentrations vs. observed MAMA Study milk concentrations
Additional modeling information
Supplemental References

First Visit Questionnaire, U.S. EPA MAMA Study (Fenton 2004)
Title of Study: Methods Advancement for Milk Analysis (MAMA)
Sponsored by: U.S. Environmental Protection Agency
Study number: 03-EPA-207 Today's Month /Year: ___________
Interviewer's Name:____________________________ Interview start time:_____________

Subject code:

Attach Consent code here once signed.

Attach Questionnaire code here and proceed with questions.

We would like you to answer the following questions for us so that we can obtain information about your lifestyle and surroundings. The subject matter should in no way alarm you, as this is just an information-gathering device to help us understand other information that is obtained from your samples. Some of the questions may have multiple answers. Please feel free to ask questions if you have any. We want you to be comfortable answering the following:

1. What is your month and year of birth? ______________

2. What is your height? __________

3. What is your weight? _________

4. What is your child's age? (round to the nearest week) ____________

5. How many children have you given birth to previously? ____________

6. How many children have you previously breastfed? _______________

7. If you have had previously breastfed a child(ren), about how long? __________

8. Were you informed at any time during your pregnancy that you had
   gestational diabetes Yes ________ No ________
   pre-eclampsia/toxemia Yes ________ No ________
   excessive weight gain Yes ________ No ________

9. How many times have you breast-fed your baby since midnight? __________

10. Is your baby being fed formula in addition to breast milk? (yes/no) _________
    How many times in a 24-hour period are you breast-feeding? ____________
    *How many times in a 24-hour period is your baby drinking formula?*________

DOI: 10.1289/EHP332

Questionnaire (Cont'd)

Title of Study: Methods Advancement for Milk Analysis (MAMA)

11. Are you currently taking any prescription medication(s) on a regular basis?
*If yes, please specify:*____

12. Have you taken any non-prescription medications in the last 24 hours?
*If yes, please specify:*____

13. Do you have any cold/flu symptoms (sore throat, runny nose, fever, sore muscles) today?
Yes ___________ *No* _________

14. Are you currently experiencing any allergy symptoms?
Yes ___________ *No* _________

15. What county do you live in and how long have you lived in this county? ___________
____

16. How long have you lived in North Carolina?_________The United States?___

What is your race? (circle one) white, black or African American, Asian, Hispanic or Latino, Native American (Indian, Alaskan, Hawaiian), mixed.

17. What is your approximate household income? (circle one)
Under $29,999 $30K to 50K $50K to 75K $75K to 100K over $100K

18. What is the highest grade of formal schooling completed?_________________

19. How long have you lived in your present dwelling?______________________

20. How old is your home or rental unit?__________________________________

21. Does your home have an enclosed garage attached?______________________

22. How old is the carpeting in your home? ______________________________
____

23. How old are any of the upholstered pieces of living room furniture in your home?____
____
____

24. Do you have vinyl blinds on your windows that are over 5-6 years old?
Yes ___________ No _________ How many?_________________

25. Do you have any lead-containing paint or asbestos in your home? (circle one)
Yes to both No Don't know Probably yes to one _________

DOI: 10.1289/EHP332

Questionnaire (Cont'd)

Title of Study: Methods Advancement for Milk Analysis (MAMA)

26. What are the average hours you spend in your home each day? ____________

27. How many hours do you spend in your vehicle each day?_________________
Make and year:____

28. Are you exposed to second-hand cigarette smoke in your workplace or at home?
Yes _____ No _________
*If yes, approximately how many hours/day are you exposed?*_______________

29. Are you presently on maternity leave from your place of employment?
Yes ___________ No ________
If you work outside the home:
*How many hours a day are you away from your home?*____________
In which county do you work? ____________________

30. Estimate how many hours a week you spend within 6 feet of a computer.
____

31. Estimate how many hours per day you spend within 6 feet of a television.
____

32. Do you pursue any of the following hobbies?

Furniture upholstery/refinishing	Yes ___No ___
Pillow making	Yes ___No ___
Painting	Yes ___No ___
Gardening	Yes ___No ___
Computer repair	Yes ___No ___
Hobbies that require the use of airplane glue or solvents	Yes ___No ___
Talk on a cellular phone more than 1 hour per day	Yes ___No ___
Talk on a cellular phone more than 3 hours per day	*Yes ___No ___*

33. How often do you: (1 = never, 2 = rarely, 3 = sometimes, 4 = often)

wear nail polish ______________	use hair styling products ________________
wear foundation ______________	consume/drink tofu or soy products
color your hair ______________	eat processed meats ___________________
take recreational drugs ________	*(sausages, hot dogs, bologna, etc)*
eat microwave popcorn________	take herbal remedies/supplements________

34. In a usual week, how many hours do you spend in these occupations, activities, or establishments?

Painting	____________
Dry cleaning	____________
Chemical Plant or Lab	____________
Plastics Fabrication	____________
Plastic or Computer Recycling	____________
Furniture Refinishing/Repair	____________
Firefighter/burn trash	____________
Carpet/insulation installation	____________
Carpet cleaning	____________
Clean houses	____________
Work with flame retardant chemicals	____________
Recycle electronic goods or plastic materials	____________
Repair computers or electronic equipment	____________
Make plastic parts	____________
Work with furniture parts, furniture coverings, or carpets	____________
Computers	____________

35. Do you eat chicken? How often in each category (per week)?
Grilled____
Baked____
Fried____

36. How often do you:
eat fish Never ______ Less than once/week ___________
Once/week ________ Twice/week _______ > Twice/week _____

List all of the kinds of fish that you eat: ______________________
____

eat beef Never ______ Less than once/week ___________
Once/week ________ Twice/week _______ > Twice/week _____

List all of the ways that you prefer to cook your beef __________
____

Title of Study: Methods Advancement for Milk Analysis (MAMA)

36. How often do you: (con't.)

eat dairy products Never ______ Two times or less/week _________
Two times/week but not daily _____ Everyday _________

drink cow's milk Never ______ Two times or less/week _________
Two times/week but not daily _____ Everyday _________

37. What kind of milk do you typically drink?
Skim ____ 2% or less fat _____ whole milk _______

38. Have you had any alcoholic drinks since you have been breast-feeding ______
If yes, how many drinks/week do you have? __________________________________

39. What is the primary source of your drinking water? (city, well, bottled water)
____

40. How much water do you drink each day (e.g. 4 large glasses, 6 small cups)
____

Thank you very much for your time. Do you have any questions for me?

Time interview **ended:**___________________

Table S1. Spearman correlation between maternal age and BFR concentrations.

Variable	Serum		Milk	
	r_s	*p* -value	r_s	*p*-value
BB-153	0.47	<0.001	0.35	<0.01
BDE-28	-0.33	<0.01	n.d.	--
BDE-47	-0.27	<0.05	-0.25	0.05
BDE-85	-0.19	0.14	-0.22	0.09
BDE-99	-0.26	<0.05	-0.21	0.10
BDE-100	-0.40	<0.001	-0.33	<0.01
BDE-153	-0.27	<0.05	-0.26	<0.05
BDE-154	-0.27	<0.05	-0.19	0.135
ΣPBDEs	-0.34	<0.01	-0.28	<0.05

n.d., not determined.
Median maternal age = 31 yr (range, 21-39 yr).
n = 63-64 individual samples.

Table S2. Spearman correlation between vehicle age and BFR concentrations.

Variable	Serum		Milk	
	r_s	*p* -value	r_s	*p* -value
BB-153	0.17	0.21	-0.30	<0.02
BDE-47	0.40	<0.01	0.43	<0.01
BDE-28	0.32	<0.02	n.d.	--
BDE-85	0.33	<0.02	0.29	<0.05
BDE-99	0.31	<0.02	0.33	<0.02
BDE-100	0.39	<0.01	0.45	<0.001
BDE-153	0.26	0.05	0.29	<0.05
BDE-154	0.37	<0.01	-0.06	0.62
ΣPBDEs	0.24	0.07	0.35	<0.01

n.d., not determined.
Median vehicle age = 5 yr (range, 0.1-11 yr).
n = 63-64 individual samples.

Table S3. Spearman correlation between home age and BFR concentrations.

	Serum		Milk	
Variable	r_s	*p* -value	r_s	*p* -value
BB-153	0.25	0.05	0.10	0.42
BDE-28	-0.35	<0.01	n.d.	--
BDE-47	-0.30	<0.05	-0.30	<0.05
BDE-99	-0.25	<0.05	-0.26	<0.05
BDE-100	-0.36	<0.01	-0.36	<0.01
BDE-153	-0.14	0.28	-0.12	0.35
BDE-154	-0.19	0.14	-0.06	0.66
ΣPBDEs	-0.25	0.05	-0.24	0.06

n.d., not determined.
Median home age = 13 yr (range, 0.5-100 yr).
n = 63-64 individual samples.

Figure S1

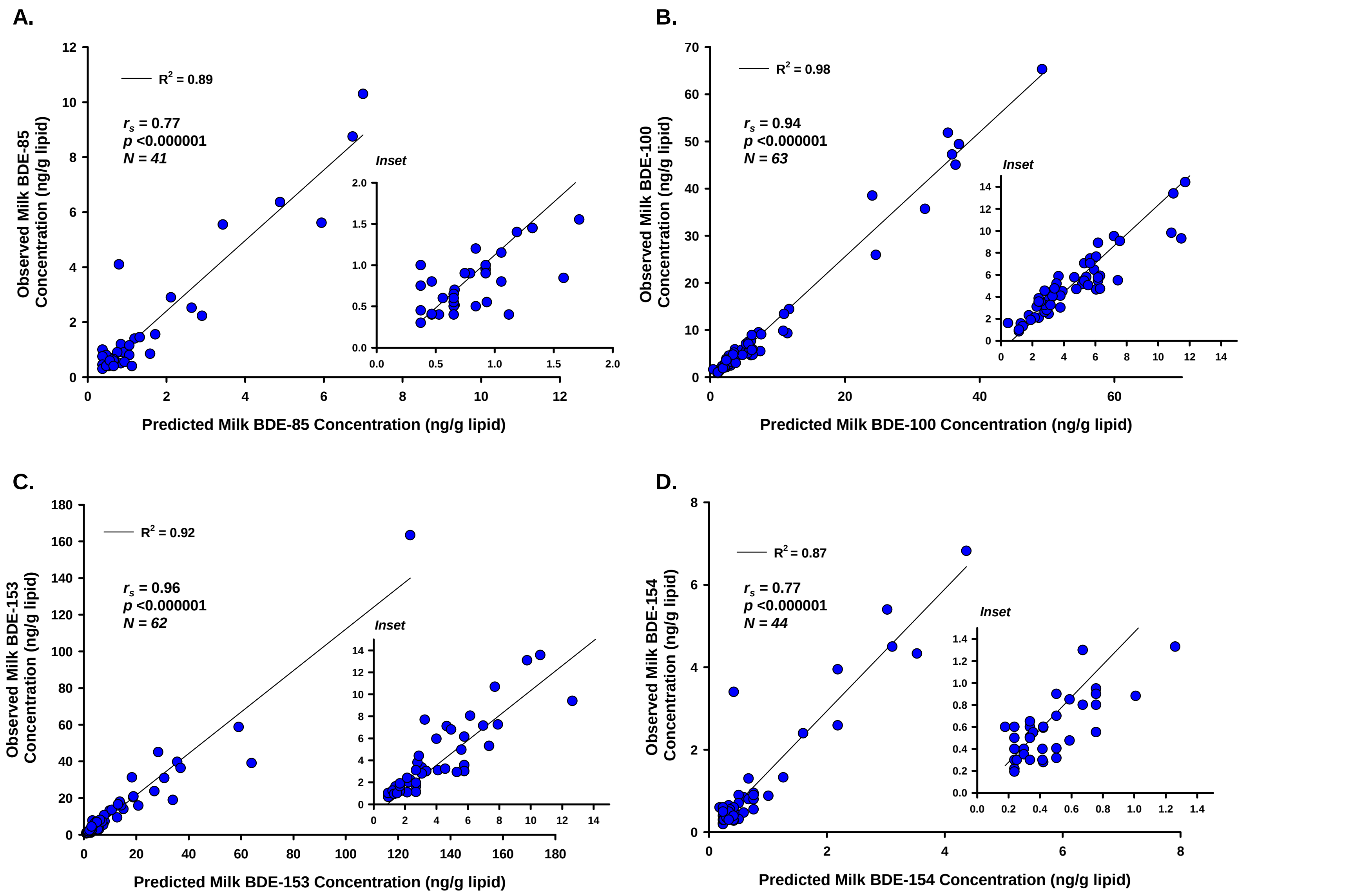

A.
R² = 0.89
rs = 0.77
p <0.000001
N = 41
Inset
Observed Milk BDE-85 Concentration (ng/g lipid)
Predicted Milk BDE-85 Concentration (ng/g lipid)
B.
R² = 0.98
rs = 0.94
p <0.000001
N = 63
Inset
Observed Milk BDE-100 Concentration (ng/g lipid)
Predicted Milk BDE-100 Concentration (ng/g lipid)
C.
R² = 0.92
rs = 0.96
p <0.000001
N = 62
Inset
Observed Milk BDE-153 Concentration (ng/g lipid)
Predicted Milk BDE-153 Concentration (ng/g lipid)
D.
R² = 0.87
rs = 0.77
p <0.000001
N = 44
Inset
Observed Milk BDE-154 Concentration (ng/g lipid)
Predicted Milk BDE-154 Concentration (ng/g lipid)

Figure S1. Predicted milk BFR concentrations vs. observed MAMA Study milk concentrations for BDE-85 (A), BDE-100 (B), BDE-153 (C), and BDE-154 (D). Milk concentrations were predicted by applying exposure models for these BDEs (Marchitti et al., 2013) to serum BDE concentrations from MAMA study participants. Solid lines are the least-squares fit of predicted and observed milk concentrations (R^2 0.87-0.98). Spearman correlation coefficients (r_s) and related p values are given for each evaluation of the relationship between predicted and observed milk concentration values (r_s = 0.77-0.96).

Additional Information for Models Previously Developed in Marchitti et al. 2013b.

Exposure models for predicting milk PBDE concentrations from serum concentrations have been previously developed (Marchitti et al. 2013b). Three U.S. studies met our study criteria (LaKind et al. 2009; Schecter et al. 2006, 2010) and only participants for whom lipid-adjusted (ng/g lipid) milk and serum PBDE concentrations where both > LOD were included. Data for seven PBDE congeners were available: BDE-28, BDE-47, BDE-85, BDE-99, BDE-100, BDE-153, and BDE-154.

Individual PBDE milk:serum partitioning ratios were calculated for each congener by dividing the milk concentration (ng/g lipid) by the serum concentration (ng/g lipid). Minimal interindividual variability was observed among participants, thus, we combined data from the three studies into one dataset. Pearson's r correlation coefficients and least-squares linear regression [SigmaPlot Systat Software, version 12.3 (2011)] were used to determine if serum and milk PBDE concentrations for each congener were significantly correlated and yielded model equations:

$$(1)\ y_i' = \beta_1 x_i$$

where y_i is the breast milk PBDE concentration of the participant ($_i$), β_1 is the slope of the regression line, and x_i is the serum PBDE concentration of the participant. β_1 provides an estimate of the congener's milk:serum partitioning ratio. The predictive power of each model was determined by *k*-fold cross-validation followed by the quantification of its predictive ability (Q^2) (Eriksson et al. 2003). Regression models for the seven PBDE congeners exhibited high predictive abilities ($Q^2 \geq 0.90$).

Supplemental References

Eriksson L, Jaworska J, Worth AP, Cronin MT, McDowell RM, Gramatica P. 2003. Methods for reliability and uncertainty assessment and for applicability evaluations of classification- and regression-based QSARs. Environ Health Perspect 111(10): 1361-1375.

Fenton SE. 2004. First Visit Questionnaire, U.S. EPA MAMA Study.

LaKind JS, Berlin CM, Jr., Sjödin A, Turner W, Wang RY, Needham LL, et al. 2009. Do human milk concentrations of persistent organic chemicals really decline during lactation? Chemical concentrations during lactation and milk/serum partitioning. Environ Health Perspect 117:1625-1631.

Marchitti SA, LaKind JS, Naiman DQ, Berlin CM, Kenneke JF. 2013b. Improving infant exposure and health risk estimates: Using serum data to predict polybrominated diphenyl ether concentrations in breast milk. Environ Sci Technol 47:4787-4795.

Schecter A, Colacino J, Sjödin A, Needham L, Birnbaum L. 2010. Partitioning of polybrominated diphenyl ethers (PBDEs) in serum and milk from the same mothers. Chemosphere 78:1279-1284.

Schecter AJ, Papke O, Harris TR, Tung KC. 2006a. Partitioning of polybromonated diphenyl ether (PBDE) congeners in human blood and milk. Toxicol Environ Chem 88:319-324.
